# Clinical features and genetic analysis of paroxysmal kinesigenic dyskinesia in children

**DOI:** 10.3389/fneur.2026.1788601

**Published:** 2026-04-09

**Authors:** Li-ping Zheng, Yun-peng Ye, Su-ping Wang, Xiao-xia Lin, Jun Hu

**Affiliations:** 1 Fujian Medical University Union Hospital, Fuzhou, China; 2 Affiliated Hospital of Putian University, Putian, China; 3 Department of Neurology, Guangzhou Women and Children’s Medical Center, Guangzhou Medical University, Guangzhou, China

**Keywords:** children, KCNMA1, mutation, paroxysmal kinesigenic dyskinesia, PRRT2

## Abstract

**Objective:**

To summarize the clinical features and genetic variation spectrum of children with paroxysmal kinesigenic dyskinesia (PKD) admitted to the Children’s Medical Center of Union Hospital Affiliated to Fujian Medical University and to provide references for clinical diagnosis and genetic counseling.

**Methods:**

A retrospective analysis was conducted on the clinical data of 6 pediatric patients diagnosed with PKD in our hospital from November 2018 to August 2025. The data included medical history, triggering factors, clinical manifestations, auxiliary examinations, and treatment responses. Whole-exome sequencing (WES) was employed to detect gene mutations, followed by Sanger sequencing for verification. The pathogenicity of the identified variants was assessed according to the guidelines of the American College of Medical Genetics and Genomics (ACMG).

**Results:**

The study cohort included 5 males and 1 female, with an age of onset ranging from 5 to 12 years. Two cases were familial, while four were sporadic. All paroxysmal episodes were induced by sudden movement, postural change, or anxiety. The clinical manifestations included unilateral or bilateral limb posturing, tremor, athetosis, dystonia, and weakness, without impairment of consciousness. The attacks were brief (duration ≤50 s) and occurred with a frequency ranging from 1-2 per month to 5-6 per day. A history of febrile seizures was present in three patients. The magnetic resonance imaging (MRI) scan of the brain of one child showed a lacune in the right frontal lobe, and video electroencephalogram (VEEG) of another child revealed abnormal epileptic discharges during the interictal period. Genetic analysis via whole-exome sequencing (WES) identified pathogenic variants in all six patients: five harbored *PRRT2* mutations (including point mutations c.649dupC, c.972delA, and c.1141delC, or exon deletions), and one had a *KCNMA1* mutation (c.946G>A). Regarding treatment, four patients administered low-dose carbamazepine and two received low-dose oxcarbazepine, all of whom achieved complete or substantial control of the dyskinetic attacks.

**Conclusion:**

The clinical features of pediatric PKD align with typical paroxysmal manifestations. The *PRRT2* gene is the primary pathogenic gene, although cases associated with *KCNMA1* mutations were also identified. Both carbamazepine and oxcarbazepine demonstrated efficacy in treating childhood PKD. Genetic testing facilitates definitive diagnosis and genetic subtyping.

## 1 Introduction

Paroxysmal kinesigenic dyskinesia (PKD) are neurological disorders characterized by brief, episodic dyskinesia triggered by sudden voluntary movements from a resting state. Clinical manifestations can include dystonia, chorea, athetosis, or ballismus, occurring with preserved consciousness and typically lasting less than 1 minute (1). The disorder often presents in childhood or adolescence, with a male-to-female ratio of approximately 2–4:1 and an estimated incidence of 1 in 150,000 (1–3). Due to its low incidence and rarity, PKD is often misdiagnosed as epilepsy or other paroxysmal neurological disorders (4). In 2011, Chinese researchers first identified *PRRT2* as the primary causative gene for PKD (5). Subsequent studies have also linked variants in genes such as *KCNMA1* to similar phenotypes (6). This study systematically analyses the clinical and genetic characteristics of six pediatric PKD patients to explore genotype–phenotype correlations and provide insights for clinical diagnosis and management.

## 2 Subjects and methods

### 2.1 Study participants

A total of six pediatric patients diagnosed with PKD at the Children’s Medical Center of Union Hospital Affiliated to Fujian Medical University from November 2018 to August 2025 were included in this study. The diagnosis was established according to the criteria proposed by Bruno et al. (1), which include episodes induced by sudden movement or postural change, an attack duration of less than 1 minute, preserved consciousness during attacks, a favorable response to anti-seizure medications, and the exclusion of other organic neurological disorders. The study was approved by the Hospital Ethics Committee (Approval no.: 2026KY006), and written informed consent was obtained from the legal guardians of all participants.

### 2.2 Data collection

Clinical data were systematically collected, including sex, age at onset, precipitating factors (e.g., sudden standing, running, or emotional stress), clinical manifestations (symptom type, duration, and frequency of attacks), past medical history (including febrile seizures or epilepsy), family history, results of auxiliary examinations (VEEG and cranial MRI), and treatment response.

### 2.3 Genetic analysis

Following informed consent from the guardians, peripheral blood samples (2 mL) were collected from the probands and their parents. Genomic DNA was extracted using a commercial kit (Thermo Fisher Scientific) according to the manufacturer’s instructions. Exonic regions were captured using the Agilent SureSelect Human All Exon V6 kit, and whole-exome sequencing (WES) was performed on the Illumina NovaSeq 6,000 platform, achieving a mean sequencing depth of ≥100× and >98% coverage at 20×. Raw sequencing data were aligned to the human reference genome (hg19) using BWA. Single nucleotide variants (SNVs) and copy number variations (CNVs) were identified using the GATK toolkit. Reference the normal population gnomAD database, variants with a population frequency <0.01% were retained for further analysis. Potential pathogenic genes were annotated using OMIM and HGMD databases. Candidate variants were validated by Sanger sequencing, with primers synthesized by Sangon Biotech (Shanghai). Sequencing results were analyzed using Chromas software. The pathogenicity classification of variants was performed according to the American College of Medical Genetics and Genomics (ACMG) guidelines (7), integrating evidence from population frequency, computational predictions (SIFT, Polyphen-2, MutationTaster), and phenotypic correlation.

### 2.4 Treatment and follow-up

According to the PKD diagnosis and treatment guidelines (8), all patients were treated with oral carbamazepine (5–10 mg·kg^−1^·d^−1^) or oxcarbazepine (10–20 mg·kg^−1^·d^−1^). Follow-up assessments were conducted at 3-month intervals to document changes in Paroxysmal dyskinesia (PxD) episodes/spells frequency and any adverse drug reactions.

### 2.5 Statistical analysis

Descriptive statistical analysis was used to analyze the data, and the clinical information of 6 children was examined. The demographic characteristics, age of onset, disease duration, clinical manifestations and treatment outcomes of the children were summarized. The count data are presented as the number of cases and percentages (%), including the distribution of disease types, gender distribution, trigger factors of attacks, and the composition of attack types; for the measurement data that do not conform to the normal distribution, the median is used to represent them, such as the age at onset and the duration of the disease; for the evaluation of therapeutic efficacy, the self-control description method is adopted to calculate the control of attacks during treatment, etc. All data are organized and analyzed descriptively using Microsoft Excel 2019 software.

## 3 Results

### 3.1 Clinical characteristics

Among the six pediatric patients with PKD, two cases were familial and four were sporadic. The cohort consisted of 5 males (83.3%) and 1 female (16.7%). The age of onset of PKD is 5–12 years, with a median of 7.5 years. Disease duration varied from 4 months to 6 years and 9 months. Precipitating factors include kinesigenic and non-kinesigenic factors, namely sudden standing (2 cases), walking (3 cases), running (1 case), general physical activity (2 cases), postural change (1 case), mental stress (1 case). Clinical manifestations were as follows: (1) Symptom type: generalized limpness (1 case), body writhing (1 case), limb weakness (4 cases), choreiform movements (1 case), athetosis (1 case), limb tremors (2 cases), gait disturbance (1 case), and falls (2 cases). (2) Attack characteristics: each episode lasted 5–40 s, with frequencies ranging from 1-2 per month to 5-6 per day. All episodes occurred without impaired consciousness. (3) Past and family history: none of the patients had a history of perinatal asphyxia, developmental delay, or intellectual impairment. Three patients had a history of febrile seizures, and two had a positive family history (Both the fathers of Patient 1 and Patient 2 had onset during childhood, and one patient’s younger sister was also affected. The father of Patient 1 reported a history of kinesigenic unilateral limb weakness during childhood. He was diagnosed with PKD and is currently free of clinical episodes on regular carbamazepine. The father of Patient 2 had a childhood history of choreiform movements and athetosis triggered by kinesigenic triggers or mental stress. He was diagnosed with PKD and remains asymptomatic on regular carbamazepine therapy). (4) Auxiliary examinations revealed the following: a cranial MRI showed a right frontal lacunar focus in one patient, with normal findings in the remainder. VEEG demonstrated sparse interictal left anterior-midtemporal epileptiform discharges in one case. No abnormalities were detected in routine blood tests, urinalysis, liver function, renal function, electrolytes, blood glucose, lactate, ammonia, or blood/urine tandem mass spectrometry (see Table 1).

**Table 1:** Clinical characteristics of the 6 pediatric patients with PKD.

Case	Gender	Age of consultation	Age of onset	Triggering factors	Clinical manifestations	Duration of each session	Seizure frequency	Past medical history	Family history	VEEG	MRI	Genetic testing	Diagnosis	Treatment	Treatment duration	Treatment response
Familial																
P1	Male	7 years old	7 years old	Stand up after prolonged sitting	Clear consciousness, soft body and trembling limbs	About 20 s	1-2 times/day	(—)	father: paroxysmal kinesigenic dyskinesia sister: infantile convulsion and paroxysmal choreoathetosis	(—)	(—)	*PRRT2 (c.649dupC)*	PKD	CBZ	4 months	Symptom control
P2	Male	10 years old	8 years old	Exercising, stand up after prolonged sitting, and mental stress	Clear consciousness, chorea, athetosis, body twisting	30–40 s	2–6 times/day or 1 time/2-3 days	Febrile convulsion	Father: paroxysmal kinesigenic dyskinesia	(—)	The right lateral ventricle is slightly larger than the left	*PRRT2 (c.972delA)*	PKD	OXC	4 years and 1 month	Symptom control
Sporadic																
P3	Female	7 years old	5 years and 1 month old	Running	Clear consciousness, weak limbs	20–30 s	1-2 times/month	(—)	(—)	(—)	Right frontal lobe cavity lesion	*PRRT2 (c.1141delC)*	PKD	OXC	6 years and 9 months	Symptom control
P4	Male	11 years and 4 months old	10 years old	Walking, running	Clear consciousness, weak left lower limb, and abnormal gait	less than 10 s	4-5 times/month	(—)	(—)	(—)	(—)	Copy number deletion variation (Chr16:29563814-30183722) × 1, including PRRT2	PKD	CBZ	4 years and 1 month	Symptom control
P5	Male	7 years and 7 months old	6 years and 1 month	Walking	Feeling premonition, clear consciousness, weakness in both lower limbs, and falling	More than 10 s	3-4 times/month or 3-4 times/day	Febrile convulsion	(—)	A small amount of interictal epileptic discharge in the left anterior middle temporal region during sleep	(—)	Copy number deletion variation (Chr16:29483682-30188588) × 1, including PRRT2	PKD	CBZ	8 months	Basic control
P6	Male	12 years old and 11 months old	11 years and 10 months old	walking, exercising, or postural change	Clear consciousness, weakness, shaking, and falling of the right limb	5-6 s	5-6 times/day	Febrile convulsion	(—)	(—)	(—)	*KCNMA1 (c.946G>A)*	PKD	CBZ	7 months	Symptom control

CBZ, carbamazepine; OXC, oxcarbazepine.

### 3.2 Genetic findings

**Figure 1:**
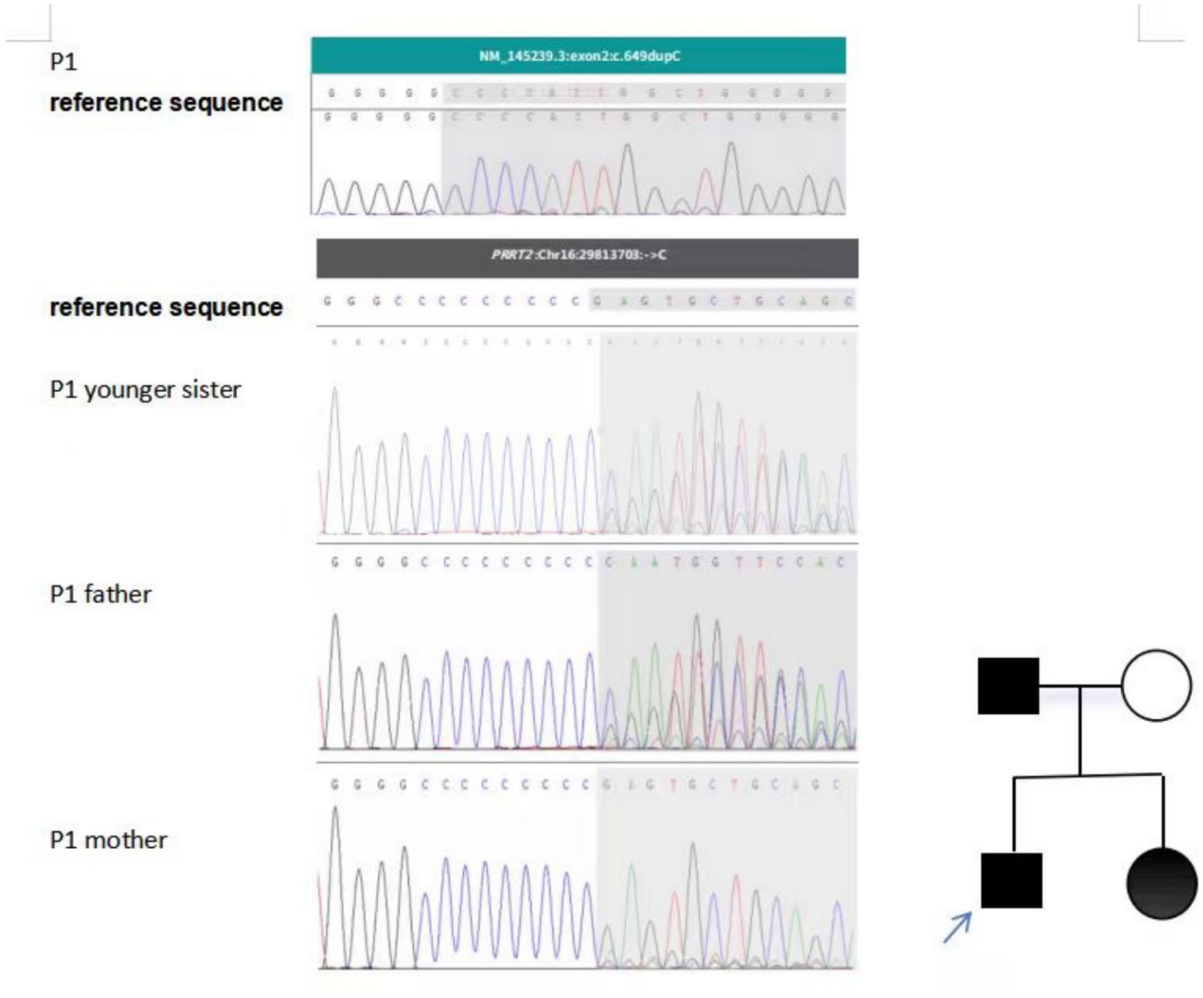
Genetic analysis and pedigree of Patient 1 (P1) with a *PRRT2* mutation. Genetic sequencing identified a c.649dupC microduplication in the *PRRT2* gene in the proband (P1). The same mutation was confirmed in his father and younger sister. The mutant allele is indicated by shaded highlighting. 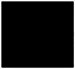 Affected male, 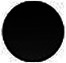 Affected female, 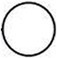 Unaffected female, Blue arrow identifies the proband.

Genetic testing revealed that Patient 1 (P1) carried a heterozygous *PRRT2* mutation (c.649dupC, exon 2), The ACMG classification of this variant is pathogenic (PVS1 + PS4), PVS1: This mutation is a frameshift mutation; PS4: The frequency of this mutation occurring in the affected population is significantly higher than that in the control population. Ebrahimi Fakhari et al. [26598493] reported that this mutation was detected in 78.5% of 1,444 patients with benign familial infantile convulsions, infantile convulsions, choreographic disorders, and motor disorders. This mutation has been included in the ClinVar database 22 times for pathogenicity and 1 time for suspected pathogenicity; consistent with a diagnosis of PKD. The same mutation was identified in P1’s younger sister, who underwent genetic testing due to infantile convulsions and paroxysmal choreoathetosis at 2 months of age. Parental sequencing showed that the father also carried the mutation (Figure 1).

**Figure 2:**
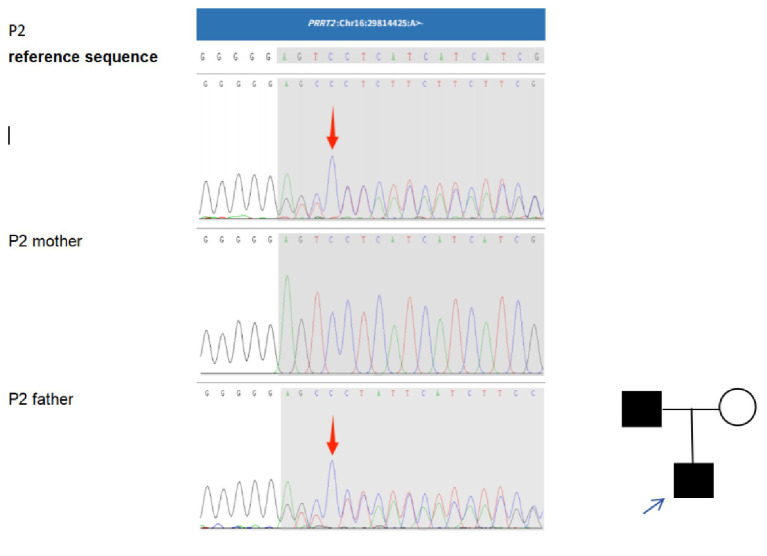
Genetic analysis and pedigree of Patient 2 (P2) with a *PRRT2* mutation: Sequencing identified a c.972delA microdeletion in the *PRRT2* gene of the proband (P2). The same variant was detected in his father. The mutant nucleotide is indicated by a red arrow. 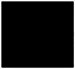 Affected male, 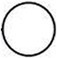 unaffected female, blue arrow identifies the proband.

Patient 2 (P2) carried a heterozygous *PRRT2* mutation (c.972delA, exon 3). The ACMG classification of this variant is clinical significance unknown (PVS1_Moderate + PM2_Supporting), PVS1_Moderate: This mutation is a frameshift mutation, located in the second to last exon, and the resulting protein sequence deletion is less than 10%. Here, PVS1_Moderate evidence is used for this mutation; PM2_Supporting: The frequency of occurrence of this mutation is not included in ExAC, gnomAD, and the Thousand Genomes Asian Population Database. The disease associated with this gene is a common autosomal dominant inheritance, and the patient is heterozygous with parents with similar medical histories at this locus. The genetic pattern can explain the patient’s illness. Parental testing confirmed that the father was also a carrier (Figure 2).

**Figure 3:**
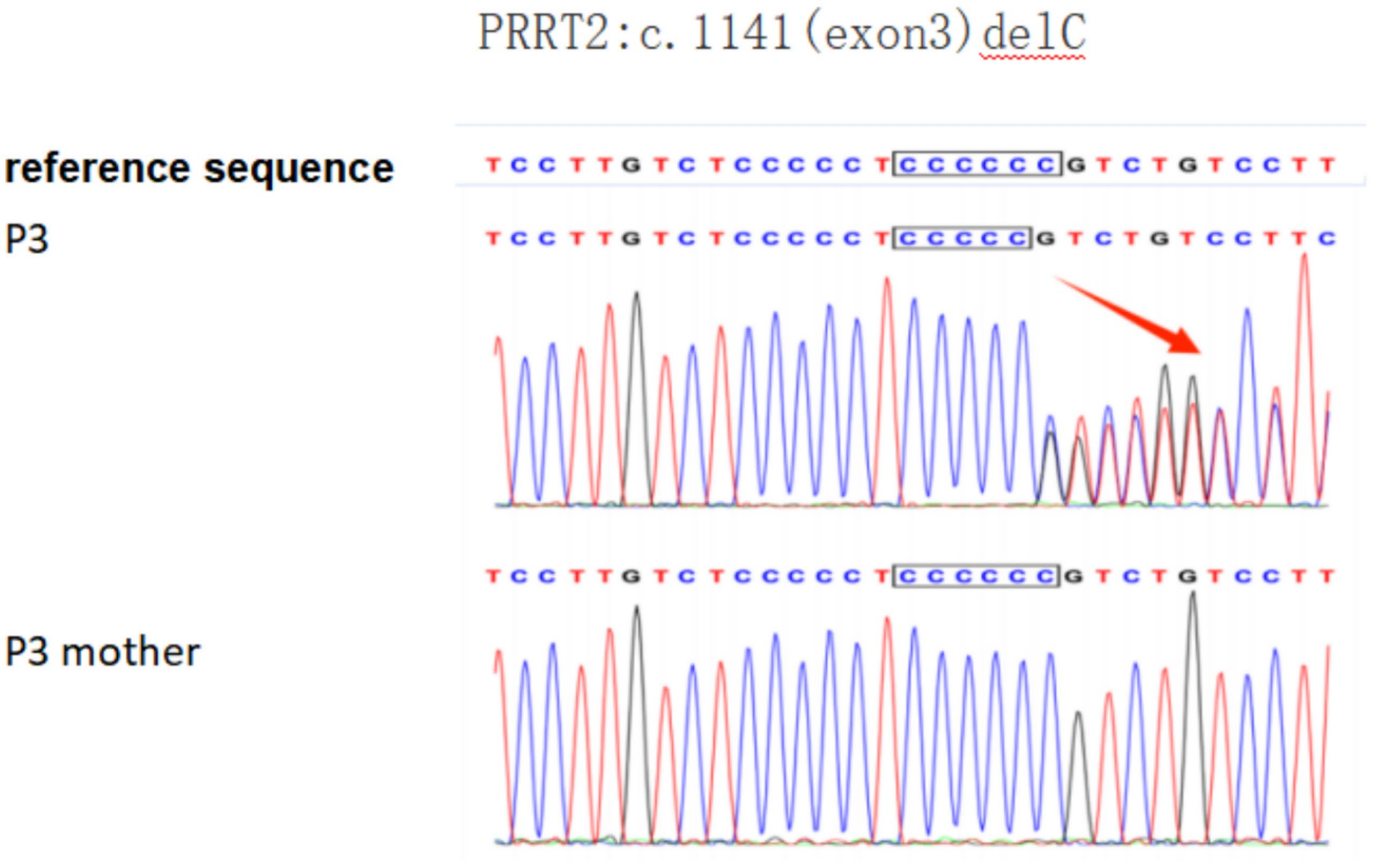
Genetic analysis of patient 3 (P3) with a *PRRT2* mutation: Sequencing identified a c.1141delC microdeletion in the *PRRT2* gene of the proband (P3). The mutant nucleotide is indicated by a red arrow.

Patient 3 (P3) carried a heterozygous PRRT2 mutation (c.1141delC, exon 3). The ACMG classification of this variant is possibly pathogenic (PVS1 + PM2), PVS1: LOF mutations may lead to loss of gene function; PM2: MAF < 0.005, Belongs to low-frequency variation. No pathogenic mutations were detected in either parent (Figure 3).

**Figure 4:**
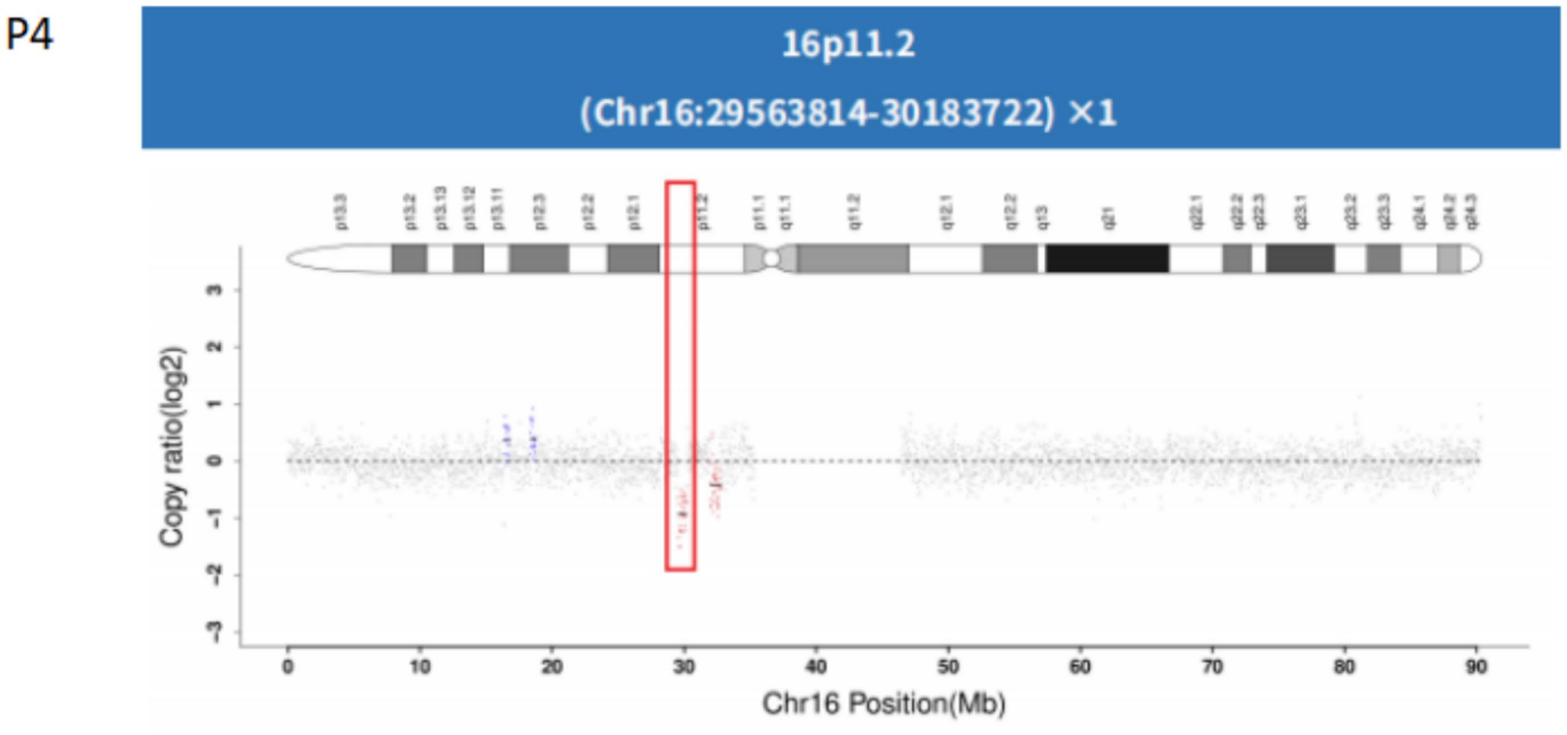
Genetic analysis of patient 4 (P4) with a *PRRT2* mutation a. Analysis identified a 0.62 Mb segmental deletion in the *PRRT2* gene of the proband (P4). The deleted region is indicated by a red box.

In Patients 4 (P4) and 5 (P5), CNV analysis identified deletions in the 16p11.2 region, measuring 0.62 Mb and 0.705 Mb, respectively, indicating pathogenic copy number variations (CNVs), Both deletions encompassed the *PRRT2* gene (Figure 4).

**Figure 5:**
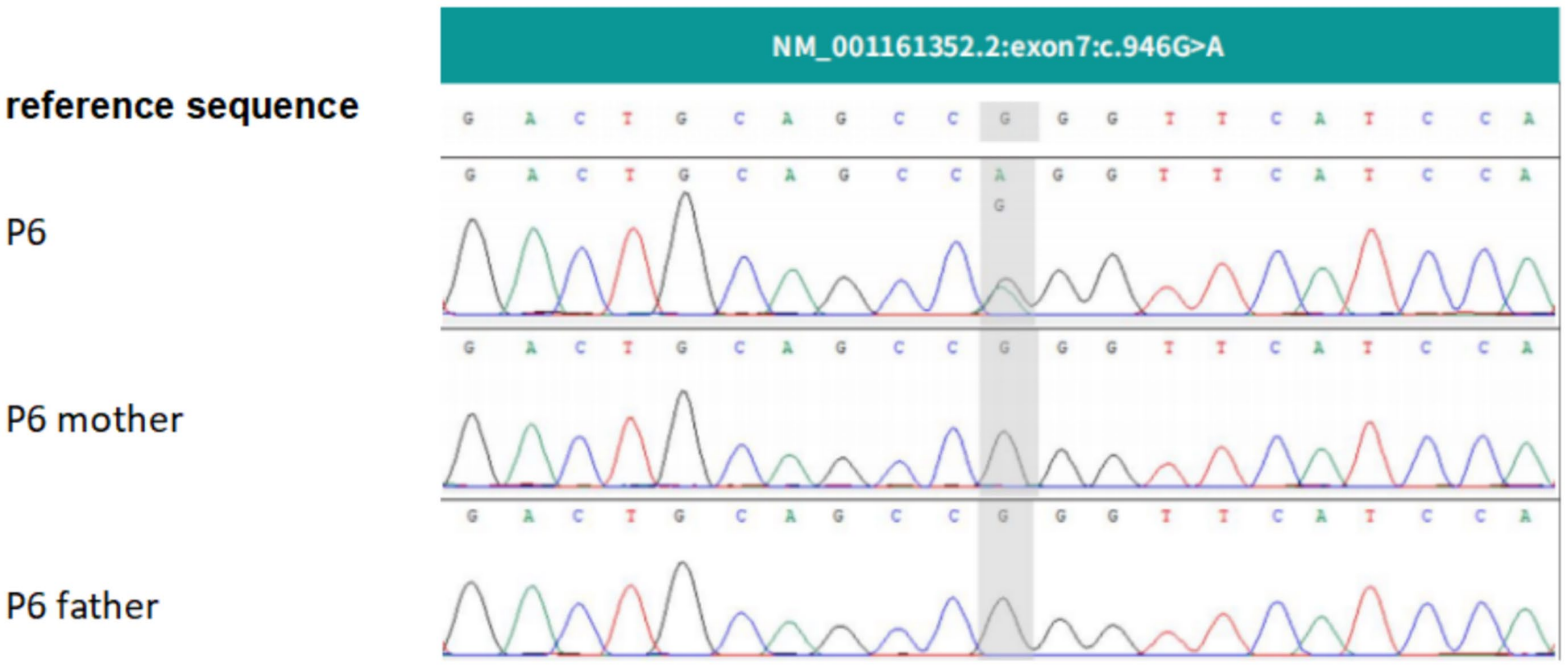
Genetic analysis of Patient 6 (P6) with a *KCNMA1* mutation. Sequencing identified a c.946G>A heterozygous mutation in the *KCNMA1* gene of the proband (P6). The mutant nucleotide is highlighted. The mutant nucleotide is indicated by gray shading.

Patient 6 (P6) carried a heterozygous *KCNMA1* mutation (c.946G>A, exon 7). The ACMG classification of this variant is clinical significance unknown (PS2-Moderate + PM2Supporting + PP3 + PP2), PS2-Moderate: This mutation detected in this study is a newly discovered mutation (the possibility of parental reproductive gland or somatic cell chimerism cannot be ruled out). The disease associated with this gene is consistent with the symptoms of the patient detected in this study, but it does not have high specificity. Therefore, there is evidence to use PS2-Moderate for this mutation. PM2-Supporting: This mutation has a very low frequency of occurrence or is not included in the gnomAD population database. PP3: Multiple mutation prediction software suggest that the mutation may be harmful. PP2: The missense variant of this gene has a high pathogenic potential, with a Z-score>3.09 included in the gnomAD database. SIFT, Polyphen_HDIV, Polyphen_HVAR, LRT, Mutation Taster, Mutation Assessor Predict harmful software, among which 5 software predict that the mutation may be harmful, Parental sequencing did not detect this variant in either parent (Figure 5).

### 3.3 Treatment course

Among the six confirmed PKD patients, four were treated with low-dose carbamazepine and two with low-dose oxcarbazepine. Attack frequency was significantly reduced after 1 month of treatment and was completely or substantially controlled after 3 months. Patient P5 continued to experience occasional breakthrough attacks and is currently under dose adjustment. No severe adverse effects were reported, with only one case of mild dizziness that did not require drug discontinuation (see Table 2).

**Table 2:** Summary of drug treatment reactions.

Patient ID	Key rationale for drug selection	Drug	Time to remission	Efficacy	Reported adverse events
P1	Older onset age	CBZ	2 weeks	Complete control	None
P2	Standard first-line treatment	OXC	1 month	Complete control	None
P3	Standard first-line treatment	OXC	2 months	Complete control	None
P4	Older onset age	CBZ	1 month	Complete control	None
P5	Standard first-line treatment attempts	CBZ	3 months	Basic control	None
P6	Older onset age	CBZ	1 month	Complete control	Mild dizziness

## 4 Discussion

Paroxysmal kinesigenic dyskinesia (PKD) is a rare neurological disorder with distinct clinical and genetic features that has attracted increasing attention in recent years. Through a comprehensive analysis of pediatric PKD cases, this study provides further insights into its clinical characteristics, genetic basis, and the correlation between them.

Regarding clinical features, PKD can be classified into primary and secondary forms. Primary PKD is further subdivided into familial and sporadic types based on the presence or absence of a family history (9), with familial PKD following an autosomal dominant inheritance pattern (10). All six PKD patients included in this study were diagnosed with the primary form, comprising two familial and four sporadic cases. PKD commonly presents during childhood and adolescence, most commonly between 6 and 16 years of age, with a significantly higher incidence in males than in females (3, 11). Attacks are commonly provoked by sudden changes in movement, such as standing up from a resting position, turning, walking, or running. Emotional factors, including fear, surprise, anxiety, or stressful situations such as crossing the street, may be associated trigger and increase attack frequency and severity (12). Some patients may experience sensory aura before an episode, such as numbness or a cold sensation in the affected limb. During attacks, patients exhibit involuntary movements of the limbs and trunk, including dystonia, choreiform movements, torsion spasms, or ballism (5), with dystonia and choreiform movements being the most frequent manifestations. Approximately 70% of patients display abnormal facial expressions or dysarthria during episodes (13). The involvement may be limited to one limb or one side of the body; it may alternate between sides or affect both sides simultaneously. The duration of attacks is brief, generally lasting from several seconds to less than 1 min, with over 98% of patients experiencing episodes shorter than 1 min (14). The frequency of attacks varies substantially among individuals, ranging from several episodes per year to dozens per day. In the initial stages of the disease, attack frequency is generally low, increases during adolescence, and gradually decreases around the age of 20 in most patients, with episodes becoming rare or ceasing entirely after the age of 30 (14, 15). Consciousness remains intact during attacks, and patients are able to communicate normally. Some patients may have a history of benign familial infantile seizures, febrile seizures, or epilepsy during early childhood. In rare cases, PKD may be accompanied by developmental delay, intellectual impairment, language deficits, or features of autism spectrum disorder (9, 16, 17).

In the present study, all six PKD patients experienced episodes triggered by actions such as standing up after prolonged sitting, turning, walking, or running. Emotional stress increased attack frequency in some cases, and a few patients reported sensory auras before onset. Clinical manifestations included dystonia and choreiform movements, with each episode lasting less than 1 min. Attack frequency varied widely among patients, from 1-2 times per month to 5-6 times per day. Consciousness was preserved during attacks, and all patients remained able to engage in coherent conversation. Some patients had a history of febrile seizures during infancy. These clinical features are consistent with the characteristic presentation of PKD.

The genetic mechanism represents a core area of PKD research. This study retrospectively analyzed clinical data and genetic testing results of six pediatric PKD patients, further validating the pivotal role of the *PRRT2* gene in PKD pathogenesis. *PRRT2* mutations were detected in five patients, including the hotspot mutation c.649dupC and novel mutations c.972delA and c.1141delC, as well as copy number deletions in the 16p11.2 region encompassing *PRRT2*. The *PRRT2* gene c.972delA is included in the HGMD database, and it has been reported in PubMed that this locus was identified in a Han male with episodic motor dysfunction. However, the *PRRT2* gene c.1141delC is not included in the HGMD and Clinvar databases, and has not been reported in PubMed. The protein encoded by *PRRT2* is primarily expressed in presynaptic neuronal membranes, participating in the regulation of neurotransmitter release and voltage-gated sodium channel function. Loss of its function may lead to abnormal neuronal excitability, thereby triggering PKD episodes (5, 18, 19). The clinical manifestations of *PRRT2*-mutated patients in this study aligned with typical PKD characteristics, and most showed favorable responses to sodium channel blockers (such as carbamazepine and oxcarbazepine), further supporting the pathological mechanism that *PRRT2*-related PKD belongs to channelopathy (20). Two patients had a family history, with phenotypic heterogeneity within the families (e.g., in P1’s family, the proband presented with PKD while the younger sister presented both seizures and paroxysmal dyskinesia and the father presented PKD in the childhood), suggesting incomplete penetrance and phenotypic variability in *PRRT2*-related disorders (9, 10).

Notably, one patient was found to carry a *KCNMA1* mutation (c.946G>A). This mutation site has not been included in the HGMD and Clinvar databases, and there have been no reports on it in PubMed. *KCNMA1* encodes the BK potassium channel, widely expressed in the central nervous system and involved in neuronal repolarization and excitability regulation. Recent studies have linked *KCNMA1* mutations to various neurological phenotypes, including epilepsy, developmental delay, and paroxysmal movement disorders (21). In this study, patient P6 presented with typical PKD and responded well to carbamazepine, this mutation site may be a new site of the PKD pathogenic gene. However, the causal relationship between *KCNMA1* and PKD requires further validation through functional studies.

This study has certain limitations: the sample size is small, the statistical power is limited, and it is difficult to conduct statistical comparisons between subgroups, which may affect the representativeness of the results. However, as PKD is a rare disease, single-center small-sample studies are a common model in clinical research. The mutations of cases P2, P3, and P6 in this study have not been verified by functional experiments, and especially the pathogenic association between *KCNMA1* mutation and PKD still requires more evidence to support. Future research should aim to expand the sample size and incorporate functional experiments and long-term follow-up to better elucidate the genotype-phenotype correlations and variability in treatment response in PKD. Such efforts will provide a more robust foundation for achieving precise diagnosis and personalized treatment.

## Data Availability

The datasets presented in this study can be found in online repositories. The names of the repository/repositories and accession number(s) can be found in the article/supplementary material.
